# Liberal Preoperative Fasting in Adults Undergoing Elective Surgery: A Scoping Review Protocol

**DOI:** 10.1155/2024/1519359

**Published:** 2024-05-08

**Authors:** Haoyue Gan, Hangcheng Liu, Huaping Huang, Mei He

**Affiliations:** ^1^School of Nursing, North Sichuan Medical College, Nanchong, Sichuan, China; ^2^Mianyang Central Hospital, School of Medicine, University of Electronic Science and Technology of China, Mianyang, Sichuan, China

## Abstract

**Background:**

Prolonged fasting before surgery has negative effects on the physiology and psychology of patients. Preoperative liberal fasting proposes that patients can drink clear liquids before entering the operating theater, challenging the guideline strategy of a two-hour preoperative liquid fast for adults. In recent years, there have been an increasing number of studies on liberal preoperative fasting in adults. However, currently there is no consensus on the safe amount of fluid consumed, adverse effects, or benefits of this new policy.

**Objective:**

This scoping review protocol will map the existing evidence of liberal preoperative fasting in adults undergoing elective surgery for clinical practice, to summarize more scientific evidence to healthcare professionals when providing perioperative care. *Methods and Analysis.* The methodology will follow the six steps of the Arksey and O'Malley methodological framework and be guided by the Preferred Reporting Items for Systematic Reviews and Meta-Analyses extension for Scoping Review. A comprehensive search of six databases will be performed from their inception to 31 May 2023 to identify suitable English studies. Two trained investigators will independently screen and extract the data, and any disagreements will be judged by a third investigator. The results of the study will be presented as graphs or tables. *Ethics and Dissemination*. This scoping review only examines literature in the database, without reference to human or animal studies, and therefore does not require ethical approval. The findings of this scoping review will be published in peer-reviewed journals or presented at conferences. *The Registration Number*. This scoping review has been registered in the Open Science Framework (https://doi.org/10.17605/OSF.IO/PMW7C).

## 1. Introduction

Preoperative fasting has always been considered by patients and physicians as a safe routine procedure for elective surgery. Since the 1940s, Mendelssohn found that anesthesia is related to the incidence of pulmonary aspiration [1]. To prevent this complication, strict preoperative fasting has been considered by anesthesiologists as a routine clinical practice [2, 3]. However, long periods of fasting can cause dehydration in patients, especially when combined with factors such as intestinal preparation, which can decrease patient blood volume, leading to low blood pressure, thirst, hunger, and even psychological discomfort such as fatigue, anxiety, fear, and despair [4–6].

The conventional fasting approach suggested that a gastric volume greater than 25 ml would increase the risk of aspiration [7], but this notion has been disproved by subsequent research, showing that gastric volume between 100 and 110 ml is typical in fasted patients [8, 9]. In 1986, Maltby et al. [10] challenged the overnight fasting routine, who found no correlation between gastric volume or pH values with the ingestion-surgery interval in patients given 150 ml of water. Aschkenasy et al. [11] also found that drinking water at different fasting times did not have a significant correlation with gastric residual fluid. Although aspiration can lead to aspiration pneumonia and ultimately death, the incidence is relatively very low at around 0.014% [12, 13]. Fluid aspiration is extremely rare in elective patients. Most cases of aspirated pneumonia are due to inappropriate anesthetic techniques [13]. Therefore, prolonged fasting to prevent aspiration seems unnecessary.

With the advancement of perioperative physiology research, it has been acknowledged that surgery, being a form of stress, can trigger specific responses in the pituitary and sympathetic nervous systems [14], causing an array of expected alterations in elevated metabolism and heightened catabolism [15]. These changes negatively affect immune function and the process of wound healing [16]. Carbohydrate loading is known to increase muscle glycogen synthesis and storage and delay the onset of high energy expenditure, reduce the stress response to surgery, and accelerate body recovery [17, 18]. The prolonged period of preoperative fasting restricted the patient's intake of all sources of energy, including carbohydrates. According to the survey, carbohydrates account for 60%–70% of total energy in the human body [19]. Therefore, restoration to normal daytime metabolism by loading carbohydrate before surgery may have an advantage over traditional fasting procedures. This perspective was confirmed by preoperative glucose infusion [20]. Oral carbohydrate drinks and intravenous injections had the same effect in reducing postoperative insulin resistance, but carbohydrate drinks could significantly reduce preoperative discomfort without affecting gastric contents [21]. Based on the above reasons, 2 h clear carbohydrate drinks before surgery are recommended for adults in the preoperative fasting guidelines [22]. Commonly used clear carbohydrate beverages containing maltodextrin, a complex carbohydrate that is easily emptied from the stomach (unlike solid food) [23], enhance perioperative nutrition in enhanced recovery after surgery (ERAS), improve patient well-being and satisfaction, and reduce the length of hospital stay [24–26]. Although guidelines for preoperative fasting have been registered for 20 years, our adherence to the recommendations is not satisfactory [27]. The primary issue is that the preoperative fasting period for patients exceeded the recommended duration of the guidelines. Several studies have reported that the fasting time for clear fluids exceeds 6 hours, with approximately 21% of patients fasting for over 12 hours [28, 29]. These findings can be explained by several factors. First, differences in operational efficiency between hospitals lead to delays or uncertainty in the surgical schedule [30]. Second, anesthetists, nurses, and surgeons do not understand the significance of the new recommendations and do not communicate or collaborate effectively [31]. Third, due to excessive concern about accidental inhalation, anesthetists still advise patients not to eat after midnight. Finally, patients with limited understanding of fasting principles were also more prone to excessive fasting [4]. Although the clear fluid fasting time decreased from midnight to two hours before surgery, the results in clinical practice are not as significant as we thought.

With the introduction of the ERAS concept, optimization and shortening of perioperative fasting plays an important role in modern surgical patient management [32]. Consequently, researchers have proposed a novel approach to managing adult perioperative fasting, advocating liberal preoperative fasting that encourages the consumption of clear fluids before patients are transferred to the operating theater. The new fasting policy has been implemented in certain patients. One hour of clear liquid fasting (including water with or without sugar, pulp-free juice, and non-milk tea or coffee) for children prior to anesthesia has been found to improve the condition of patients and is recommended by guidelines [33–37]. However, the fasting strategy for adults has not been relaxed according to the guidelines. Recent studies suggest that a permissive approach to fasting may be appropriate for adults, as adults have a similar physiology to children [35, 38]. The current multidisciplinary agreement maintains that patients with no or low risk of aspiration are allowed to consume clear fluids without restrictions until sedation [35]. According to a study, patients who abstained from drinking within 2 hours before surgery had higher rates of postoperative nausea and vomiting within 24 hours (5.2% and 2.8%, respectively) compared to those who were allowed to drink until surgery (3.8% and 2.2%, respectively) [39]. In another preoperative liberal fasting study, patients were allowed to consume 150 ml of clear fluids every hour before entering the operating room, which not only reduces the incidence of postoperative nausea and vomiting but also benefits patient satisfaction [40]. The above evidence presented indicates that liberal preoperative fasting for adults is feasible. For fasting practice, these protocols provide a specific operational element, that is, the concept of unrestricted drinking allows the patient to consume clear fluids continuously during a day of surgery, before being asked to enter the operating room, and thus is not limited by the flexibility of surgical schedule. After the implementation of the new policy, the duration of fluid fasting was substantially shortened to approximately 2-3 hours, and less postoperative discomfort for adults occurred [40, 41]. However, the study by Atkinson et al. [42] found no harm to patients from the liberal fasting policy, but it did not show clear benefits. Therefore, we want to synthesize more evidence to investigate the impact of unrestricted fasting on adults before surgery.

Currently, there is no consensus on the amount of fluid intake during liberal preoperative fasting or on the advantages and disadvantages of this practice. It is also unclear what healthcare providers and patients think about the new strategy. We did not identify any ongoing or completed systematic reviews or scoping reviews on this topic in our initial database search. Therefore, this scoping review will adopt a wider perspective to determine the significance, consensus, and controversial areas of liberal preoperative fasting in adults undergoing elective surgery.

## 2. Methods and Analysis

### 2.1. Study Designs

This scoping review will be used to map the literature on the subject of “Preoperative liberal fasting in adults undergoing elective surgery.” Results will be presented according to the extension of Preferred Reporting Items for Systematic Reviews and Meta-Analyses for Scoping Review (PRISMA-ScR) [43]. This scoping review will be carried out using the framework suggested by Arksey and O'Malley [44].

### 2.2. Research Questions

1. What is the research trend for preoperative liberal fasting in adults?
2. What is the safe range volume of clear fluid for liberal preoperative fasting in adults?
3. What are the adverse effects and benefits of preoperative liberal clear fluid fasting for adults?
4. What is the current state of awareness among patients and healthcare professionals about liberal clear fluid fasting in adults before surgery?
5. What can current research on liberal preoperative fasting in adults tell us about managing the perioperative period and implementing ERAS protocols?
6. What future research will be needed on preoperative liberal fasting in adults?

### 2.3. Search Strategies

The search strategy will aim to identify studies on this topic through an initial search of PubMed (Table 1). The text words in the titles and abstracts of relevant articles, along with the index terms used to describe the articles, were utilized to devise a comprehensive search strategy for Medline, Scopus, Embase, CINAHL, PsycINFO, Web of Science, and the Cochrane Library from their inception to 31 May 2023. We will manually identify more additional evidence from the reference lists of screened and eligible articles, and an updated search will be performed prior to the final analysis. All references will be organized, managed, and collected from each database and stored using EndNote 20.0 (Philadelphia, PA, USA), a bibliographic software program [45].

### 2.4. Selecting Studies

Team members collaboratively develop the inclusion and exclusion criteria for the study. The criteria for inclusion of a study in the scoping review were as follows: (1) population—patients were over 18 years; (2) interventions—study participants underwent liberal preoperative fasting for elective surgery; (3) outcomes—any outcomes; and (4) study design—reviews or intervention studies. Exclusion criteria were as follows: (1) containing incomplete data; (2) publication language other than English; and (3) conference abstracts and web pages. We will include commentary articles, as they are often complementary to original studies.

According to the above criteria, two trained researchers (GHY and HHP) independently read the title and abstract of the article. They then review the full text to verify and confirm the eligibility criteria, following the same process. The kappa value will be calculated to determine the extent of inter-rater agreement regarding study inclusion. In the event of disagreement between the two investigators during these two steps, a third investigator (HM) will assist and resolve the disagreement. The results of the search and the inclusion process of the study will be plotted as a flowchart, showing details of included and excluded studies at each selection stage.

### 2.5. Data Charting

The data extraction framework, customized to the study objectives, is created jointly by the team members. The details of the framework include title, author, year of publication, country, study design, preoperative fasting approach, and main outcomes. The information will be recorded in Microsoft Excel. The research data were extracted independently by two researchers (GHY and HHP). The study authors will be contacted for missing or supplementary data if necessary.

### 2.6. Collating, Summarizing, and Reporting Results

Insights into preoperative liberal fasting information in adults will be gained from the analysis of the collected data. To present a comprehensive and objective overview of the findings, the extracted data from the included literature will be displayed graphically as diagrams or tables. Consistent with the objectives of this study, we will provide an overview of liberal fasting in surgical adult patients, the population of patients, the types of studies included, the time of research, and the effects of fasting on patients. Quantitative (e.g., results) and qualitative (e.g., content and thematic analysis) methods will be used to synthesize the findings and other data from the selected studies to provide a narrative summary of the results. The data aggregation, analysis, and reporting process will follow the PRISMA-ScR guidelines for specialized evaluations.

## 3. Discussion

Our scoping review aims to obtain knowledge about the existing evidence and the feasibility of introducing liberal fasting methods into clinical practice and to offer suggestions for further research in this area. When examining existing unrestricted fasting protocols used in hospitals, it is plausible to identify potential areas that have not yet been fully explored. We will investigate areas of agreement in the treatment of liberal fasting in adults, as well as areas where further research and the creation of new protocols are necessary to generate innovative concepts for perioperative management and new approaches to rapid recovery of patients.

## Figures and Tables

**Table 1:** Initial search strategy in PubMed.

Step	Strategy
#1	“Preoperative Period” [Mesh] OR “Preoperative Care” [Mesh]
#2	(((liberal fluid) OR (unrestricted fluid)) OR (free fluid)) OR ((liberal fasting) OR (unrestricted fasting)) OR (free fasting))
#3	((((“Elective Surgical Procedures” [Mesh])) OR (elective surgery)) OR (surgery)) OR (“Enhanced Recovery After Surgery” [Mesh])
#4	(“Adolescent” [Mesh]) OR “Child” [Mesh]
#5	(Children [Title/Abstract]) OR (Teenager [Title/Abstract])
#6	#4 OR #5
#7	#1 AND #2 AND #3 NOT #6

## Data Availability

The datasets collected and analyzed in this study are available from the corresponding authors upon reasonable request.
